# Phenolic metabolites, biological activities, and isolated compounds of *Terminalia muelleri* extract

**DOI:** 10.1080/13880209.2017.1406531

**Published:** 2017-11-27

**Authors:** Walaa A. El-Kashak, Samir M. Osman, Ahmed H. Gaara, Sayed A. El-Toumy, Tahia K. Mohamed, Iñaki Brouard

**Affiliations:** a Chemistry of Natural Compounds Department, National Research Centre, Cairo, Egypt;; b Department of Pharmacognosy, Faculty of Pharmacy, October-6-University, 6th October City, Egypt;; c Department of Chemistry, College of Science, Jazan University, Jazan, Saudi Arabia;; d Chemistry of Tannins Department, National Research Centre, Cairo, Egypt;; e Instituto de Productos Naturales y Agrobiología, La Laguna, Tenerife, Spain

**Keywords:** DPPH, antioxidant, anticancer, molecular docking

## Abstract

**Context:***Terminalia muelleri* Benth. (Combretaceae), is rich with phenolics that have antioxidant and cytotoxic activities. No screening studies were published before on *T. muelleri*.

**Objective:** The study focused on isolation and identification of secondary metabolites from aqueous methanol leaf extract of *T. muelleri* and evaluation of its biological activities.

**Materials and methods:** The *n*-butanol extract was chromatographed on polyamide 6, and eluted with H_2_O/MeOH mixtures of decreasing polarity, then separated by different chromatographic tools that yielded 10 phenolic compounds. The antioxidant activity of the extract was evaluated by investigating its total phenolic and flavonoid content and DPPH scavenging effectiveness. The extract and the two acylated flavones were evaluated for their anticancer activity towards MCF-7 and PC3 cancer cell lines. Molecular docking study of the acylated flavones was performed against topoisomerase enzyme.

**Results and discussion:** Two acylated flavonoids, apigenin-8-C-(2″-*O*-galloyl) glucoside **1** and luteolin-8-C-(2″-*O*-galloyl) glucoside **2**, were isolated and identified for the second time in nature, with eight tannins (**3**–**10**), from the leaves of *T. muelleri*. The extract and compound **10** showed the most significant antioxidant activity (IC_50_ = 3.55 and 6.34 μg/mL), respectively. The total extract and compound **2** demonstrated cytotoxic effect against MCF-7 with IC_50_ = 29.7 and 45.2 μg/mL respectively, while compound **1** showed cytotoxic effect against PC3 (IC_50_ = 40.8 μg/mL). The docking study of compounds **1** and **2** confirmed unique binding mode in the active site of human DNA topoisomerase enzyme.

**Conclusions:***Terminalia muelleri* is a promising medicinal plant as it possesses high antioxidant activity and moderate cytotoxic activity against MCF-7.

## Introduction

For centuries to date, herbal medicines have been treating both human and animal diseases worldwide while various pharmacological studies continue to validate their uses (Fahmy et al. 2015). Those studies focus on a variety of biological activities that include antioxidant, antibacterial, anti-inflammatory and cytotoxic activities. Phenolics are safe natural antioxidants (Farnsworth and Bingel 1977), as they retard the progress of many diseases by protecting the body from free radicals. Although many studies have already been undertaken on a number of medicinal plants for their antioxidant activity (Kumaran and Karunakaran 2007), great interest in searching for new plant sources of antioxidant activity continues (Göktürk Baydar et al. 2007; Prakash et al. 2007). The continual use of the Egyptian plants in folk medicine (Abdel-Azim et al. 2011; Omara et al. 2012), for treatment of various disorders, prompted the researchers to carry out intensive studies on these plants. One of these Egyptian plants is *Terminalia* (Combretaceae), which comprises 200 species in tropical and subtropical regions around the world (de Morais Lima et al. 2012). *Terminalia* genus ranks amongst the most widely used plants for folk medicine (Cock 2015). As such *Terminalia* species have wide pharmacological actions such as are antioxidant (Lin et al. 1997; Cheng et al. 2003; Lee et al. 2005; Pfundstein et al. 2010), antiviral (El Mekkawy et al. 1995; Valsaraj et al. 1997), antimicrobial (Elizabeth 2005), hepatoprotective (Marzouk et al. 2002; Eesha et al. 2011), antihyperlipidemic, antidiabetic (Latha and Daisy 2011), anti-inflammatory (Nair et al. 2010) and cytotoxic activities (Ponou et al. 2010). Some *Terminalia* species also possess wound healing properties (Dougnon et al. 2014). Different secondary metabolites such as triterpenes, tannins, flavonoids, phenolic acids, lignan and lignan derivatives were reported from *Terminalia* species (Lee et al. 2007; Eloff et al. 2008; Pfundstein et al. 2010; Fahmy et al. 2015). Originated in Australia, *T. muelleri* Benth. possesses some good antimicrobial activity against *Staphylococcus aureus*, methicillin-resistant *S. aureus*, *Escherichia coli* and *Candida albicans* (Anan et al. 2010; Silva and Serramo 2015). The presence of a strong antibacterial activity of *T. muelleri* is attributed to gallic acid while its antioxidant activity is due to a high phenolic content (Fahmy et al. 2015). The main purpose of this study is to investigate, for the first time, the chemical and biological constituents of *Terminalia muelleri* as there are no screening studies on this plant species. Molecular docking has been carried out to assist with understanding the anticancer activity of the isolated compounds of *T. muelleri* and explore the binding mode of flavonoids to topoisomerase enzyme.

## Materials and methods

### General methods

Nuclear magnetic resonance (NMR) were performed on a Bruker AMX 500 instrument (Billerica, MA) with standard pulse sequences operating at 500 MHz in ^1^H NMR and 125 MHz in ^13^C NMR, chemical shifts are given in *δ* values (ppm) using tetramethylsilane as the internal standard and DMSO-*d*_6_ as solvent at room temperature and coupling constants were reported in Hz. Infra-red (IR) spectra were recorded with Nexus 670 FT-IR FT-Ramen spectrometer (Gaithersburg, MD) as potassium bromide discs. UV spectra were recorded with Shimadzu UV-1601 (Kyoto, Japan). HRESI-MS was taken on a Micromass Autospec (70 eV) spectrometer. Column chromatography was performed on polyamide 6 and Sephadex LH-20.

### Plant material

The leaves of flowering *T. muelleri* were collected in 2013 from the Zoo garden (Giza, Egypt). The plant species was identified by Dr. M. El-Gibaly, Lecturer of Taxonomy and Consultant for Central Administration of Plantation and Environment. A voucher sample (No.: TM # 0610) was deposited at Department of Pharmacognosy, Faculty of Pharmacy, October-6-University, Egypt.

### Extraction, isolation and purification

Powder of the air-dried leaves of *T. muelleri* (1 kg) was defatted with CHCl_3_ (3 × 1 L) and extracted with CH_3_OH:H_2_O [7:3 (5 × 3 L)] at room temperature. The combined extracts were filtered, evaporated under reduced pressure and lyophilized (150 g). The dry extract (100 g) was redissolved in 2 L with distilled H_2_O and extracted with *n*-butanol (5 × 2 L). After evaporation of solvents, the *n*-butanol extract and the remaining H_2_O phase gave dark brown solids 30 and 50 g, respectively. The *n*-butanol extract was loaded on a polyamide 6 column chromatography (50 × 3 cm). The column was eluted with H_2_O, and then H_2_O–MeOH mixtures of decreasing polarity and 10 fractions (1 L, each) were collected. The major phenolic fractions obtained were combined into five fractions after chromatographic analysis using paper chromatography. Fraction I (3.5 g) was fractionated by column chromatography on Sephadex LH-20 with aqueous EtOH (0–70%) for elution to give compounds **1** (35 mg) and **7** (25 mg). Fraction II (2.5 g) was subjected to column chromatography on cellulose and *n*-BuOH saturated with H_2_O as an eluent to give two major subfractions, then each of them was separately fractionated on a Sephadex LH-20 to yield pure samples **2** (30 mg) and **5** (18 mg). Using the same procedure fraction III (2.2 g) gave chromatographically pure samples **3** (15 mg) and **4** (38 mg). Fraction IV (1.5 g) was chromatographed on Sephadex LH-20 using aqueous acetone (0–25%) for elution to give pure sample **6** (20 mg). Fraction V (1.4 mg) has been separated on Sephadex LH-20 CC using *n*-butanol-water saturated to give three compounds which were further purified on Sephadex LH-20 CC using MeOH:H_2_O (1:1) to give pure samples of **8** (18 mg), **9** (20 mg) and **10** (25 mg).

#### Apigenin 8-C-(2″-O-galloyl) glucoside 1

Yellow amorphous powder; UV *λ*_max_ (MeOH) nm: 267, 332; IR (KBr) *υ*_max_ cm^−1^: 1612, 1654, 3409; ^1^H NMR (500 MHz, DMSO-*d*_6_) *δ* (ppm): 8.12 (2H, d, *J* = 8.7 Hz, H-2′,6′), 6.94 (2H, *J* = 8.7 Hz, H-3′,5′), 6.82 (1H, s, H-3), 6.75 (2H, s, H-2‴,6‴), 6.10 (1H, s, H-6), 4.95 (1H, d, *J* = 10.1, Hz, H-1″), 5.49 (1H, t, *J* = 9.80, 9.35 Hz, H-2″), 3.83 (1H, dd, H_a_-6″), 3.62 (1H, m, H_b_-6″), 3.59 (1H, m, H-3″), 3.54 (1H, t, *J* = 9.30, 8.95 Hz, H-4″), 3.38 (1H, m, H-5″).^13^C NMR (125 MHz, DMSO-*d*_6_) (see Table 1).

#### Luteolin 8-C-(2″-O-galloyl) glucoside 2

Yellow amorphous powder; UV *λ*_max_ (MeOH) nm: 271, 345; IR(KBr) *υ*_max_ cm^−1^: 1614, 1651, 3422; ^1^H NMR *δ* (ppm): (500 MHz, DMSO-*d*_6_) 7.64 (1H, d, *J* = 8.3, 2 Hz, H-6′), 7.56 (1H, d, *J* = 2 Hz, H-2′), 6.91 (2H, *J* = 8.3 Hz, H-5′), 6.75 (2H, s, H-2‴,6‴), 6.68 (1H, s, H-3), 6.10 (1H, s, H-6), 4.95 (1H, d, *J* = 10.10 Hz, H-1″), 5.49 (1H, t, *J* = 9.80, 9.40 Hz, H-2″), 3.86 (1H, dd, H_a_-6″), 3.64 (1H, dd, H_b_-6″), 3.60 (1H, m, H-3″), 3.52 (1H, dd, *J* = 9.45, 8.95 Hz, H-4″), 3.40 (1H, m, H-5″). ^13^C NMR (125 MHz, DMSO-*d*_6_) (see Table 1).

**Table 1.** ^13^C NMR data of compounds **1** and **2** in DMSO-*d*_6_ at 125 MHz.

Atom no.	**1**	**2**
Aglycone moiety		
2	164.02	164.14
3	102.39	102.35
4	181.96	181.88
5	161.17	160.55
6	97.68	97.67
7	162.05	162.07
8	103.82	103.81
9	156.39	156.40
10	102.47	102.42
1′	121.60	122.01
2′	129.02	114.06
3′	115.85	145.83
4′	160.55	149.66
5′	115.85	115.71
6′	129.02	119.46
Glucose moiety		
1″	71.07	71.12
2″	72.24	72.22
3″	75.97	76.10
4″	70.56	70.80
5″	82.01	82.18
6″	60.97	61.36
Galloyl moiety		
1‴	119.34	119.41
2‴,6‴	108.53	108.57
3‴,5‴	145.22	145.22
4‴	138.14	138.13
7‴	164.79	164.78

### Determination of total phenolic and flavonoids contents

The concentration of total phenolics of the plant extract and fractions was determined according to the method described by Kumar et al. (2008), gallic acid was used as standard. Briefly, a mixture of 100 µL of plant extract (100 µg/mL), 500 µL of Folin–Ciocalteu reagent and 1.5 mL of Na_2_CO_3_ (20%) was shaken and diluted up to 10 mL with water. After 2 h, the absorbance was measured at 765 nm using a spectrophotometer. All determinations were carried out in triplicate. The total phenolic concentration was expressed as gallic acid equivalents (GAE). Total flavonoid concentration of plant extract and fractions was determined according to the reported procedure (Kumaran and Karunakaran 2007). One hundred microlitres of plant extract (10 mg/mL) in methanol was mixed with 100 µL of 20% AlCl_3_ in methanol and a drop of acetic acid, and then diluted to 5 mL with methanol. The absorbance was measured at 415 nm after 40 min against the blank. The blank consisted of all reagents and solvent without AlCl_3_. All determinations were carried out in triplicate; the total flavonoid concentration was expressed as rutin equivalents (RE).

### Radical scavenging activity towards DPPH

The 1,1-diphenyl-2-picrylhydrazyl is a stable deep violet radical due to its unpaired electron. In the presence of an antioxidant radical scavenger, which can donate an electron to DPPH, the deep violet colour decolourize to the pale yellow non-radical form (Ratty et al. 1988). The change in colourization and the subsequent fall in absorbance are monitored spectrophotometrically at 520 nm. In a flat bottom 96-well microplate, a total test volume of 200 µL was used. In each well, 20 µL of different concentrations (6.25–100 µg/mL final concentration) of tested samples were mixed with 180 µL of ethanolic DPPH and incubated for 30 min at 37 °C. Triplicate wells were prepared for each concentration and the average was calculated. Then photometric determination of absorbance at 520 nm was performed by microplate ELISA reader. The half maximal scavenging capacity (SC_50_) values for each tested sample and ascorbic acid was estimated via dose curve and was calculated using the curve equation.

### Measurement of potential cytotoxicity by SRB assay

Potential cytotoxicity was tested using the method of Skehan et al. (1990). Cells were plated in 96-multiwell plate (10^4^ cells/well) for 24 h before treatment with the tested samples to allow the attachment of cells to the wall of the plate. Different concentrations of the tested samples (0, 1, 2.5, 5 and 10) were added to the cell monolayer, triplicate wells were prepared for each individual dose. Monolayer cells were incubated with the tested samples for 48 h at 37 °C and in atmosphere of 5% CO_2_. After 48 h cells were fixed, washed and stained with Sulfo-Rhodamine-B stain. Excess stain was washed with acetic acid and attached stain was recovered with Tris EDTA buffer. Finally, the colour intensity was measured in an ELISA reader. The relation between surviving fraction and drug concentration is plotted to get the survival curve of each tumour cell line after the specified compounds. IC_50_ was calculated as the mean of triplicate determinations ± standard deviation, concentrations in µg/mL.

### Docking

Docking simulation study has been performed in the CADD Lab. (Dept. of Medicinal Chemistry; Faculty of Pharmacy; October 6 University) using Molecular Operating Environment (MOE^®^) version 2014.09, Chemical Computing Group Inc. (Montreal, Canada). The computational software operated under ‘Windows XP’ installed on an Intel Pentium IV PC with a 1.6 GHz processor and 512 MB memory.

### Target compounds optimization

The target compounds were constructed into a 3D model using the builder interface of the MOE program. After checking their structures and the formal charges on atoms by 2D depiction, the target compounds were subjected to a conformational search. All conformers were subjected to energy minimization, all the minimizations were performed with MOE until a RMSD gradient of 0.7823 kcal/mol and RMS distance of 0.1Å´ with MMFF94X force-field and the partial charges were automatically calculated. The obtained database was then saved as MDB (Molecular Data Base) file to be used in the docking calculations.

### Optimization of the enzymes active site

The X-ray crystallographic structures of human DNA topoisomerase I (70 kDa) complexed with camptothecin and covalent complex with a 22 base pair DNA duplex (Code: 1T8I) were obtained from RCSB-Protein data bank. The enzyme was prepared for docking studies by: (i) adding hydrogen atoms to the system with their standard geometry, (ii) checking the atoms connection and type for any errors with automatic correction, (iii) fixation of the selection of the receptor and its atoms potential and (iv) using MOE Alpha Site Finder for the active site search in the enzyme structure using all default items. Dummy atoms were created from the obtained alpha spheres.

### Docking of the target molecules to human DNA topoisomerase I (code: 1T8I) active sites

Docking of the selected conformer in database of the target compounds was done using MOE-Dock software. The enzyme active site file was loaded and the Dock tool was initiated. The program specifications were adjusted to dummy atoms as the docking site, triangle matcher as the placement methodology and London dG adjusted to its default values as the scoring methodology to be used and was adjusted to its default values. The MDB file of the ligand to be docked was loaded and Dock calculations were run automatically, finally the obtained poses were studied and the poses showed best ligand–enzyme interactions were selected and stored for energy calculations.

## Results and discussion

### Structural elucidation

**Figure 1.**
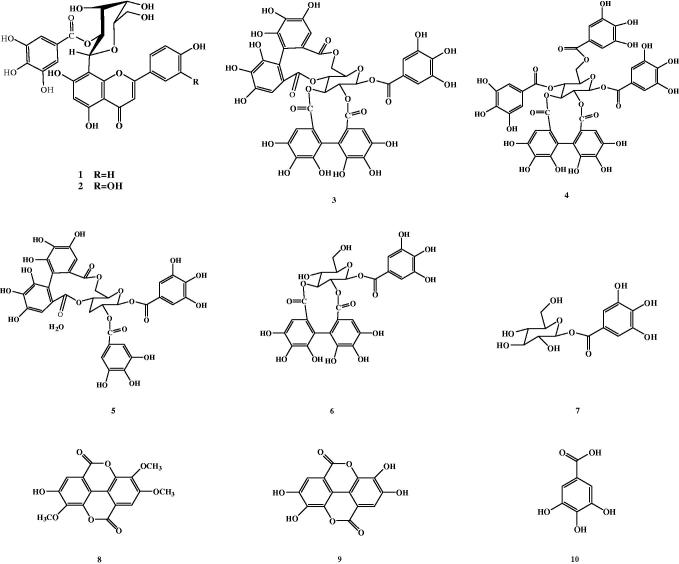
Chemical structures of isolated compounds of *Terminalia muelleri*.

**Figure 2.**
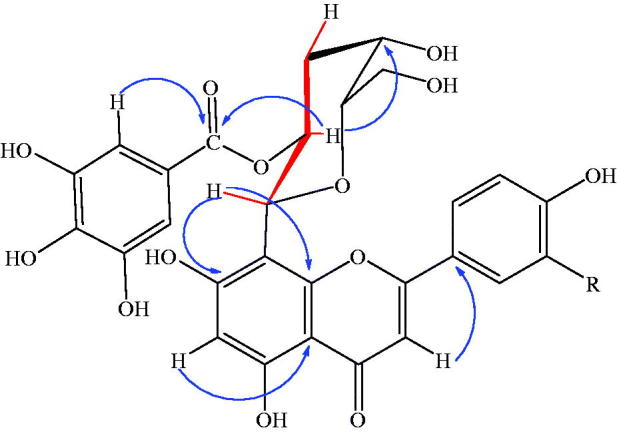
[GRAPHIC] HMBC correlations for 1 and 2. [GRAPHIC] Cosy correlations for H-2″ and H-1″ & 3″.

Two galloylated flavonoids and eight tannins were separated from the alcoholic extract of *T. muelleri* leaves for the first time using different chromatographic tools (Figure 1). Compound **1** was isolated as yellow amorphous powder that displayed a dark purple spot on paper chromatogram under UV light, which turned to yellow color when fumed with ammonia vapor, as well as, giving a characteristic color reaction (lemon yellow) with Naturestoff reagent. UV spectral data of **1** was measured and showed that it has a flavone nature (Mabry et al. 1970; Harborne et al. 1975). The molecular formula of compound **1** was estimated as C_28_H_24_O_14_ with a pseudo molecular ion at *m/z* 607.1057 [M + Na]^–^. The IR spectrum showed carbonyl, aromatic and hydroxyl bands. ^1^H NMR spectrum of compound **1** showed an AA′ BB′ system at *δ* 8.12 and 6.94 ppm for *p*-disubstituted benzene ring, in addition to, two singlets at *δ* 6.82 for H-3 and 6.10 for H-6 indicated the presence of apigenin structure, which is substituted at position 8. The appearance of one sharp singlet at *δ* 6.75 ppm is for the two magnetically equivalent protons of the galloyl group. In the aliphatic region, the appearance of a doublet at *δ* ppm 4.95 is attributed to the anomeric proton of the sugar with large *J* value ≥9 Hz of β-C-glycoside. The conspicuous downfield of the triplet signal of proton 2″ of the glucose moiety at *δ* 5.49 is attributed to the attachment of the gallic acid (Liu et al. 1997; Rayyan et al. 2010) and the downfield of the anomeric proton confirmed that that the substitution is at 2″. ^13^C NMR spectrum of compound **1** was very close to those for apigenin-8-C-glycoside as shown in Table 1 (Markham and Chari 1982), the spectrum showed a peak at *δ* 103.82 due to the glycosylation of the aglycone at C-8 (Agrawal and Bansal 1989), in addition to a singlet at *δ* 181.96 assigned to the carbonyl carbon. Twenty-eight carbon signals, one methylene, 13 methine and 14 quaternary carbons are shown in ^13^C DEPT experiments, and further confirmation is achieved by HSQC experiment for all carbons carrying protons. The site of attachment of the sugar is confirmed by the observed cross peak between the anomeric proton appeared at *δ* 4.95 and carbon 8 at *δ* 103.82 in the HMBC spectrum, indicating that attachment is at C-8, while the attachment of gallic acid to the sugar moiety is confirmed by a remarkable cross peak between proton 2″ at *δ* 5.49 and the carbonyl carbon of gallic acid at *δ* 164.79. Depending upon all mentioned data, **1** was elucidated to be apigenin 8-C-(2″-*O*-galloyl) glucoside. Compound **2** has the same physical properties as **1** except in the color reaction as it gave an orange color with Naturestoff reagent as it has two hydroxyl groups in B ring. The molecular formula of compound **2** was estimated as C_28_H_24_O_15_ from the HRESI-MS with a pseudo molecular ion at *m/z* 623.1019 [M + Na]^–^. The chemical structure of compound **2** was very similar to that of **1**; in ^1^H NMR the most significant difference between them was the ABX system for B substituted benzene ring that appeared at *δ* ppm 7.64, 7.56 and 6.91 for protons 6′, 2′ and 5′, respectively, indicating the presence of luteolin moiety. ^13^C NMR spectrum of compound **2** is illustrated in Table 1 which was very close to those for luteolin-8-C-glycoside (Agrawal and Bansal 1989).^13^C DEPT experiments revealed the presence of one methylene, 12 methine and 15 quaternary. Based upon spectral data, compound **2** was confirmed to be luteolin 8-C-(2″-*O*-galloyl) glucoside. HMBC and COSY correlations of compounds **1** and **2** are shown in Figure 2. Compounds **1** and **2** are isolated for the second time from natural source (Latté et al. 2002). The known compounds were identified as 1-*O*-galloyl-2,3,4,6-dihexahydroxydiphenoyl-β-d-glucopyranoside (**3**), 1,4,6-tri-*O*-galloyl-2,3-hex ahydroxydiphenoyl-β-D-glucopyranoside **4**, 1,2-di-*O*-galloyl-4,6-hexahydroxydiphenoyl-β-d-glucopyranoside (**5**), 1-*O*-galloyl-2,3,hexahydroxydiphenoyl-β-d-glucopyranoside (isostrictinin) (**6**), 1-*O*-galloyl-β-d-glucopyranoside (**7**), 3,3′,4,tri-*O*-methylellagic acid (**8**), ellagic acid (**9**) and gallic acid (**10**), by using different spectroscopic analysis and comparing with previously reported data (Nonaka et al. 1989; Abdulladzhanova et al. 2003; Grace et al. 2014).

### Evaluation of antioxidant activity

The free radical scavenging activity of the alcoholic extract and the isolated compounds were evaluated on DPPH (2,2-diphenyl-1-(2,4,6-trinitrophenyl)hydrazinyl). The total extract and compounds **2**, **9** and **10** exhibited potent antioxidant scavenging activity towards DPPH, with IC_50_ values of 3.55, 56.32, 67.54 and 6.34 µg/mL, respectively, while the other compounds exhibited moderate activity (Table 2), using ascorbic acid as a positive control. The DPPH^•^ radical scavenging activity is known to be related to the nature of phenolics contributing to their electron transfer/hydrogen donating ability (Brand-Williams et al. 1995). As previously reported, the antioxidant activity of phenolic compounds depends on their skeleton and the number of hydroxyl groups and their arrangement (Bouchet et al. 1998), where the *ortho* dihydroxy systems of B ring increase radical scavenging activity (Rice-Evans et al. 1996). The higher H-donating ability of the free hydroxyl groups, explained the higher radical scavenging activity of the isolated compounds (Natella et al. 1999). The total flavonoidal and phenolic content of the extract of *T. muelleri* leaves was found to be 276.48 ± 28.07 and 162.09 ± 16.67 mg/mL, respectively.

**Table 2.** DPPH radical scavenging activity of the total extract and the isolated compounds.

Compound	DPPH free radical scavengingactivity IC_50_^a^ (µg/mL)
Alcoholic extract	3.55 ± 0.20
Compound **1**	150.02 ± 2.84
Compound **2**	56.32 ± 1.86
Compound **3**	198.56 ± 2.68
Compound **4**	189.42 ± 2.58
Compound **5**	146.24 ± 2.43
Compound **6**	126.05 ± 1.89
Compound **7**	158.46 ± 2.34
Compound **8**	118.62 ± 1.98
Compound **9**	67.54 ± 1.86
Compound **10**	6.34 ± 0.18
Ascorbic acid	40.20 ± 0.40

a Values of IC_50_ were calculated as the mean of triplicate determinations ± standard deviation, concentrations in µg/mL required scavenging the DPPH radical (100 μg/mL) by 50%.

### Anticancer activity of the acylated compounds and the total extract

**Figure 3.**
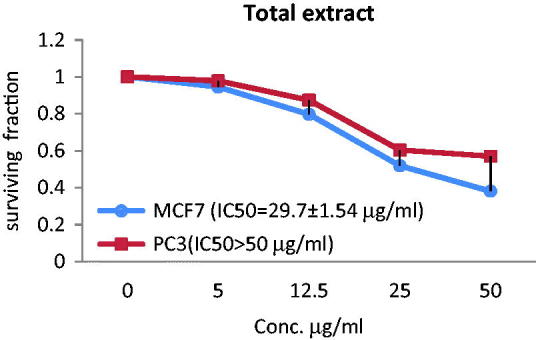
The correlations between different concentrations of the total extract of *T. muelleri* and the surviving fraction of MCF-7 and PC3 cancer cells.

**Figure 4.**
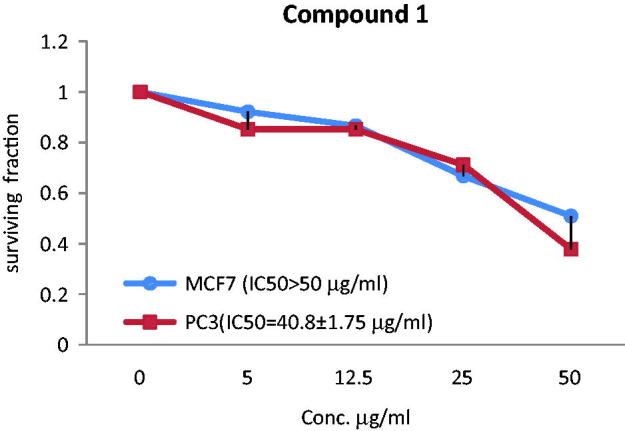
The correlations between different concentrations of compound 1 of *T. muelleri* and the surviving fraction of MCF-7 and PC3 cancer cells.

**Figure 5.**
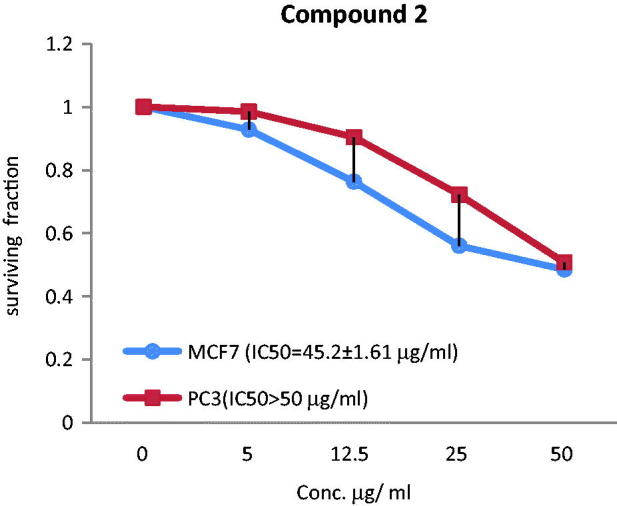
The correlations between different concentrations of compound 1 of *T. muelleri* and the surviving fraction of MCF-7 and PC3 cancer cells.

The anticancer effect of the total extract and two acylated flavonoids against MCF-7 and PC3 cancer cell lines was evaluated and expressed by median inhibitory growth concentration (IC_50_) using doxorubicin as a standard (IC_50_ = 5.10 µg/mL for PC3 and IC_50_ = 3.83 µg/mL for MCF-7). The extract showed the most significant effect against MCF-7 displaying IC_50_ value of 29.7 ± 1.54 µg/mL. Compound **2** showed moderate anticancer activity towards MCF-7 (IC_50_ = 45.2 ± 1.61 µg/mL), while the activity was diminished for **1** as IC_50_ > 50 µg/mL. On the other hand, compound **1** demonstrated antiprostate cancer activity against PC3 with IC_50_ value of 40.8 ± 1.75 µg/mL, while the extract and compound **2** showed lower activity towards PC3 with IC_50_>50 µg/mL (Figures 3–5).

### Molecular docking of compounds 1 and 2

**Figure 6.**
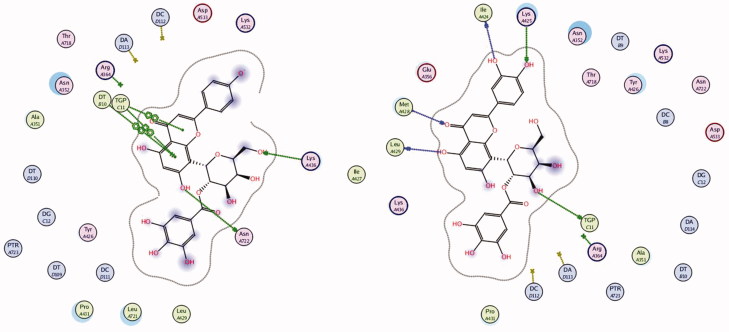
Ligand interaction representation of compounds 1 and 2 of *T. muelleri* with human topo I–DNA complex.

**Figure 7.**
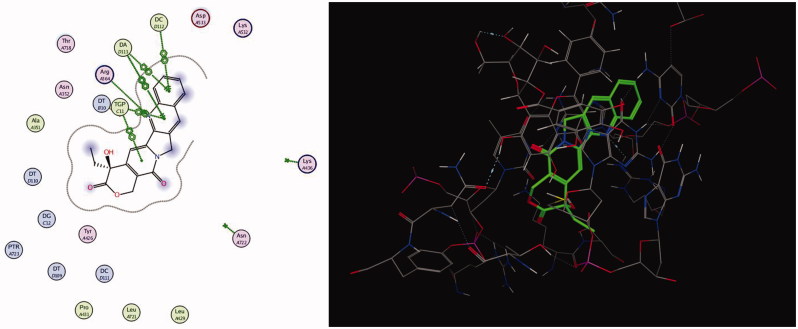
Ligand interaction representation (left) of camptothecin with human topo I–DNA complex and the corresponding 3D form (right).

**Figure 8.**
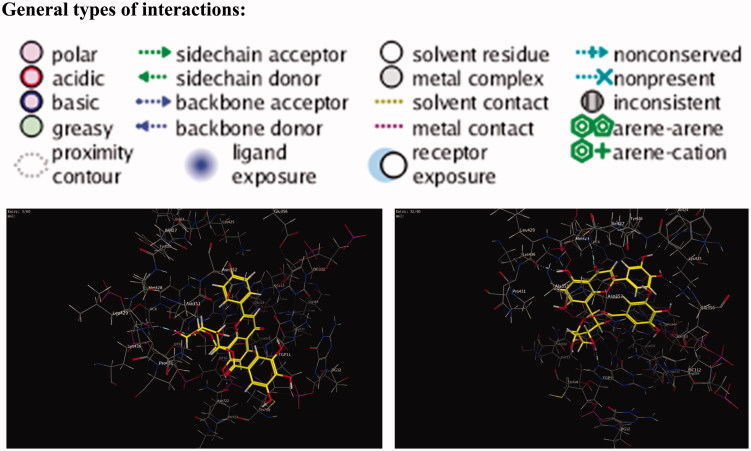
3D representation of compounds **1** and **2** of *T. muelleri* with human topo I–DNA complex.

The polyphenolic compounds present richly in human diet introduce several chemo-preventative agents (Fresco et al. 2006). Many flavonoids undergo redox chemistry including oxidative reactions, but their mechanism of action on topoisomerase is still not well defined (Bandele and Osheroff 2007), despite many performed studies. It has been proposed that genistein acts as traditional poison and that (–)-epigallocatechin-3-gallate acts as a redox-dependent poison (Bandele et al. 2008). The isolated compounds **1** and **2** are related to this class of polyphenolic compounds. *In vitro* anticancer activity of the two compounds has been determined on MCF-7 and PC3 cell lines using SRB assay. The results illustrate good activity of these compounds. Moreover, the antioxidant activity of the compounds using DPPH method illustrates pronounced effect. Based on the aforementioned observation, a molecular docking experiment has been carried out to assist understanding the anticancer activity of the isolated compounds. Molecular docking of compounds **1** and **2** was performed on human DNA topoisomerase I (PDB: 1T8I) enzyme and their interactions are illustrated in Figure 6. Using London dG force, docking of camptothecin, as the co-crystallized ligand, was performed to study the scoring energy (S), amino acid interactions and root mean standard deviation (RMSD), which enables to carry out precise validation of the docking protocol. Force field energy was used to elaborate the outcome. Camptothecin binds in the active site pocket with S = –20.0331 kcal/mol and RMSD = 0.7823. Camptothecin interacts with the amino acid ASP 533 by hydrogen bonds, the hydrogen bond is 3.27Å´. In addition, several pi–pi interactions between the aromatic rings of the ligand and the DNA residues of the target are displayed in Figure 7. The isolated compounds **1** and **2** were docked on the enzyme/DNA complex active site and the docking scores, amino acids interactions are shown in Table 3. The compounds bound to the active site of human DNA topoisomerase I enzyme (PDB: 1T8I) with energy scores –28.64 and –30.81 kcal/mol, respectively, exceeding the co-crystallized ligand camptothecin. The results indicate that there is correlation between the number of phenolic (OH) groups and the binding score. Ligand interactions and 3D representations of compounds **1** and **2** are illustrated in Figure 8.

**Table 3.** Binding scores and amino acid interactions of the docked compounds on the active site of human DNA topoisomerase I enzyme (PDB: 1T8I).

Cpds	RMSD	E-refine	Active site interaction	Interaction type	Distance
Camptothecin	0.782	–20.03			
**1**	1.697	–28.64	ASN 722	H-donor	3.28
			LYS 436	H-acceptor	2.94
			DT 10	pi–pi	3.90
			TGP 11	pi–pi	3.91
			TGP 11	pi–pi	3.73
**2**	1.5249	–30.81	LEU 429	H-donor	2.89
			TGP 11	H-donor	2.65
			ILE 424	H-donor	3.20
			LYS 425	H-acceptor	3.21
			MET 428	H-acceptor	2.81

## Conclusions

*Terminalia muelleri* is a promising medicinal plant as it exhibited potent antioxidant scavenging activity towards DPPH. Our study tends to support the therapeutic value of this plant as antioxidant drug. It demonstrated moderate cytotoxicity against MCF-7 cell line. The acylated compounds confirmed unique binding mode in the active site of human DNA topoisomerase enzyme.
